# Osteosarcoma transcriptome data exploration reveals STC2 as a novel risk indicator in disease progression

**DOI:** 10.1186/s12920-023-01456-4

**Published:** 2023-02-20

**Authors:** Ziyue Wang, Zixin Zeng, Feng Gao, Ziwei Gui, Juan Du, Ningning Shen, Yangwei Shang, Zhiqing Yang, Lifang Shang, Rong Wei, Wenxia Ma, Chen Wang

**Affiliations:** 1 Basic Medical school of ShanXi Medical University, Tai Yuan city, ShanXi Province China; 2 Department of Pathology, The Second Hospital of ShanXi Medical University, No.382 WuYi Road, 030000 Tai Yuan City, ShanXi Province China; 3 Department of Pathology, Beijing Shijitan Hospital, Capital Medical University, Beijing, China; 4 Department of Orthopedics, The Six Clinical Medical School of ShanXi Medical University, Tai Yuan, ShanXi Province China

**Keywords:** Osteosarcoma, STC2 gene, Bioinformatic analysis, Risk indicator, Molecular precise medicine

## Abstract

**Background:**

Osteosarcoma has been the most common primary bone malignant tumor in children and adolescents. Despite the considerable improvement in the understanding of genetic events attributing to the rapid development of molecular pathology, the current information is still lacking, partly due to the comprehensive and highly heterogeneous nature of osteosarcoma. The study is to identify more potential responsible genes during the development of osteosarcoma, thus identifying promising gene indicators and aiding more precise interpretation of the disease.

**Methods:**

Firstly, from GEO database, osteosarcoma transcriptome microarrays were used to screen the differential expression genes (DEGS) in cancer comparing to normal bone samples, followed by GO/KEGG interpretation, risk score assessment and survival analysis of the genes, for the purpose of selecting a credible key gene. Further, the basic physicochemical properties, predicted cellular location, gene expression in human cancers, the association with clinical pathological features and potential signaling pathways involved in the key gene’s regulation on osteosarcoma development were in succession explored.

**Results:**

Based on the selected GEO osteosarcoma expression profiles, we identified the differential expression genes in osteosarcoma versus normal bone samples, and the genes were classified into four groups based on the difference level, further genes interpretation indicated that the high differently level (> 8 fold) genes were mainly located extracellular and related to matrix structural constituent regulation. Meanwhile, module function analysis of the 67 high differential level (> 8 fold) DEGS revealed a 22-gene containing extracellular matrix regulation associated hub gene cluster. Further survival analysis of the 22 genes revealed that STC2 was an independent prognosis indicator in osteosarcoma. Moreover, after validating the differential expression of STC2 in cancer vs. normal tissues using local hospital osteosarcoma samples by IHC and qRT-PCR experiment, the gene’s physicochemical property revealed STC2 as a cellular stable and hydrophilic protein, and the gene’s association with osteosarcoma clinical pathological parameters, expression in pan-cancers and the probable biological functions and signaling pathways it involved were explored.

**Conclusion:**

Using multiple bioinformatic analysis and local hospital samples validation, we revealed the gain of expression of STC2 in osteosarcoma, which associated statistical significantly with patients survival, and the gene’s clinical features and potential biological functions were also explored. Although the results shall provide inspiring insights into further understanding of the disease, further experiments and detailed rigorous clinical trials are needed to reveal its potential drug-target role in clinical medical use.

**Supplementary Information:**

The online version contains supplementary material available at 10.1186/s12920-023-01456-4.

## Background

Osteosarcoma has been the most common primary malignant tumor rising from bone affecting firstly children and adolescents (average age 15 ~ 19) and secondly over 60 years old elderly [1]. The incidence of worldwide osteosarcoma is around 1.7–3.3 per million annually, although the incidence is relatively low, the degree of malignancy is threateningly high. The 5-year survival rate of osteosarcoma is 60 − 70%, and the 5-year survival rate of patients with recurrence or distal metastasis is less than 20% [2]. More threateningly, no notable improvement has been received for neither non-metastatic nor metastatic osteosarcoma patients survival over the past 30 years [3]. For resectable lesions, surgery has been the first choice, and for unresectable lesions, cytotoxic drugs based neoadjuvant chemotherapy and adjuvant chemotherapy combining with radiotherapy were the main treatment strategy [4].

Despite the promising effects that have been received in many other cancers for instance non-small cell lung cancer, breast cancer, colorectal and gastric cancer by immunotherapy and molecular targeting therapy [5–9], the success has not been replicated in the treatment of osteosarcoma partly attributing to the high tumor heterogeneity, which has been a major characteristic of osteosarcoma, both at the intra-tumoral level and between individuals [10]. As for the intra-tumor heterogeneity, the complexity of the somatic genome is the major cause, making it complex to identify the genome initial biological processes that drive the cancer development and explore molecular targeted therapies.

With the rapid advancing of molecular pathology technologies, inspiring improvement has been achieved in digging the genetic events in osteosarcoma development benefiting deeper understanding of the disease. In 1996, Miller CW.et al detected the mutations of TP53, RB1 and MDM2 genes in osteosarcoma by PCR-SSCP (single strand conformation polymorphism) [11], which genes were later proved to be the most frequently mutated genes in osteosarcoma, including TP53 and RB1 with the incidence of 31 − 82% and 19 − 64% in osteosarcoma respectively [12]. In 2015, Lisa Mirabello. et al. reported the effect of germline TP53 mutation on osteosarcoma genetic susceptibility [13] and germline NFIB variation on osteosarcoma metastasis [14]. In 2017, Sam behjati. et al. discovered the gene variation of insulin-like growth factor signaling pathways in osteosarcoma [15]. In 2018, Wang Di. et al. developed a study on the heterogeneity and clonal evolution of osteosarcoma, which result showed that the heterogeneity between osteosarcoma (primary and metastatic) was more significant than that within osteosarcoma, and comparing to primary lesions, the metastatic lesions have stronger immunogenicity, higher cancer antigen load, higher expression level of PD-L1 and higher level of tumor infiltrating lymphocytes [16].

Meanwhile, many other reports also discovered inspiring genetic events in the development of osteosarcoma, for example, the expression of heat shock protein p72 was reported to be related with the patient adjuvant chemotherapy effect [17], and Rb gene loss [18] as well as Her2/neu gene gain of expression indicated worse patients survival. Moreover, some genetic events, such as chromosome copy number variation (CNV), KDR, PDGFRA and VEGFA amplification, also have a high frequency in osteosarcoma [19, 20].

However, the current information are still woefully lacking comparing to the highly heterogeneous, complicated and progressive osteosarcoma nature, the common genomic initiating biological processes driving osteosarcoma development have not been defined, and no medicable gene targets have been developed yet, making it urgent for worldwide researchers to keep exploring the genetic events in osteosarcoma development thus aiding more precise understanding of the disease and identifying potential gene targets [3].

In the study, public datasets, local hospital tissues with complete clinical medical information and multiple bioinformaic analysis were combine used to explore osteosarcoma data for identifying promising prognosis indicators and potential gene targets. Firstly, we used GEO osteosarcoma data to identify the differential expression genes in osteosarcoma comparing to normal bone samples, then divided the genes into different groups based the difference level and used GO/KEGG to interpret the gene groups separately including the cellular locations, biological functions and signaling pathways the genes were mainly enriched in, followed by further function module analysis of the genes to identify promising gene clusters. Considering the fact that the main characteristic of esteosarcoma diagnosis is the formation of specific osteoid matrix [21], we focused on a 22-genes containing gene cluster which was indicated by module analysis to be associated with cancer extracellular matrix regulation. And further survival analysis of the 22 genes assisted us for identifying a key gene that potentially works as an independent prognosis indicator. Further, the clinical pathological significance of the key gene was analyzed using public dataset and the changed expression in osteosarcoma versus normal tissues was validated with local hospital tissues. Moreover, the physicochemical property, the potential biological function and probable signaling mechanisms behind the key gene’s regulation on osteosarcoma development were preliminary explored. The results shall provide inspiring insights into deeper understanding of osteosarcoma development and benefit unearthing of potential prognosis indicators.

## Materials and methods

### Data source: osteosarcoma transcriptome profiles from GEO database

From GEO online database, we widely screened the cDNA expression profiles for exploring the differential expression genes in osteosarcoma comparing to normal bone samples. The selection criteria were set as: (1) profiles were based on human samples, not animal models; (2) samples type was human osteosarcoma solid tissues or validated cancer cell lines; (3) the type of profiles data were osteosarcoma mRNA/ cDNA/ transcriptome sequencing data; (4) containing both osteosarcoma and normal control samples.

Based on above selection criteria, we picked four profiles GSE12865 [22], GSE42352 [23], GSE16088 [24] and GSE28424 [25] from GEO online database for further analyzing the differential expression genes (DEGS) in osteosarcoma comparing to normal bone samples. And of the four profiles, GSE12865 was based on GPL6244 platform [HuGene-1_0-st] Affymetrix Human Gene 1.0 ST Array [transcript (gene) version], containing 12 osteosarcoma and 2 normal human osteoblasts samples. GSE42352 was based on GPL10295 platform Illumina human-6 v2.0 expression beadchip and contains 84 osteosarcoma biopsy samples as well as 3 normal osteoblast samples. GSE16088 was based on GPL96 platform [HG-U133A] Affymetrix Human Genome U133A Array and contains 14 osteosarcoma and 3 normal samples. Meanwhile, GSE28424 was based onGPL13376 platform Illumina HumanWG-6 v2.0 expression beadchip and contains 19 osteosarcoma cell lines and 4 normal bone control samples (Table S1).

### Datasets processing: select the differential expression genes in osteosarcoma comparing to normal bone samples

After the four cDNA profiles being downloaded from GEO database, GEO2R, which was provided paired with the profiles online was in succession used to screen differential expression genes in osteosarcoma. Given the heterogeneity of osteosarcoma between individuals, we analyzed the genes in four GEO profiles separately.

All the differential expression genes in each profile were identified respectively with the criteria set as adjusted P value < 0.05 followed by being classified into 4 groups based on the |log2FC| value. To be more clear, the first group with |log2FC|<1, namely the expression discrepancy level of this group of genes was < 2 fold in osteosarcoma comparing to normal samples. The second group with 1≤|log2FC|<2, meaning the expression discrepancy level was 2 ~ 4 fold in osteosarcoma vs. normal samples. The third group with 2≤|log2FC|<3, that is to say the expression discrepancy level of this group of genes between osteosarcoma and normal was 4 ~ 8 fold. And the fourth group with |log2FC|≥3, to wit, the discrepancy level was ≥ 8 fold in osteosarcoma comparing to normal samples.

### GO/KEGG interpretation of the differential expression genes

To further understand the variation of different groups of genes functions, Gene ontology analysis (GO) and Kyoto Encyclopedia of Genes and Genomes (KEGG) [26] were used to preliminary interpret the < 2 fold, 2 ~ 4 fold, 4 ~ 8 fold and > 8 fold group of genes in four profiles separately, including their cellular locations, main biological processes, molecular functions and envolved signaling pathways. The differences and potential connections between not only 4 gene groups but also 4 different profiles were preliminary deduced. Considering the fact that more changed the genes expressions are in cancer versus normal controls, they are more likely to be validated using immunochemistry (IHC) experiment which has been one of the most commonly used tests in clinical pathology diagnosis, we mainly focused on the > 8 fold group of genes for further analysis.

### Protein-Protein Interaction (PPI) network construction and function module analysis of the genes

To next step search the interaction between different genes and explore their potential association with clinical osteosarcoma development, the protein-protein interaction (PPI) network of all the > 8 fold group of genes was constructed using Search Tool for the Retrieval of Interacting Genes (STRING) [27]. Based on the PPI network, we explored the function modules (gene clusters sharing similar function) of the genes by Molecular Complex Detection (MCODE) plug-in of Cytoscape3.6.0 software [28], and identified the top 3 gene modules for further deeply interpretation. Considering the fact that the main characteristic of osteosarcoma is the formation of specific osteoid matrix, we mainly focused on the gene modules that were related to the extracellular matrix constituent construction.

### Survival analysis of module genes to identify the credible key genes

After the genes module analysis, an inspiring module which was indicated by genes interpretation to be associated with extracellular matrix constituent regulation was highlighted, and each one of the module genes was orderly brought for univariate survival analysis using TARGET database profiles [29]. Then the genes that were indicated to be statistical significantly correlate with both osteosarcoma overall survival (OS) and disease-free survival (DFS) by univariate survival analysis would be identified as promising prognostic indicators during osteosarcoma development. The association between gene indicator expression and osteosarcoma clinical pathological features was subsequently analyzed.

### Basic physicochemical properties analysis of the key genes

Before detailed experimental validation of key genes’ differential expression and potential regulation on osteosarcoma development using local hospital tissue microarrays, GeneCards [30], which has been a widely used online knowledgebase for interpreting certain gene’s information for instance the gene’s aliases, gene domains, currently known related disorders, reported associated drugs as well as the genomic, transcriptomic, proteomic and clinical data related with the gene was used to assistant the understanding of the key gene’s basic knowledge.

Further, ProtParam [31] and ProtScale [32] which have been two effectively used online service for computing certain proteins’ physical and chemical parameters were used to analyze the key genes’ basic properties including heir theoretical isoelectric point, molecular weight, aminoacid composition, estimated protein half life, protein instability index, hydrophobicity and hydrophilicity.

### Cellular location prediction of key genes

Immunohistochemistry (IHC) has been one of the most common and widely used technologies in clinical pathological diagnosis, considering the feasibility of further clinical medical use of the gene indicators by IHC detection, multiple bioinformatic tools were used to predict the potential cellular location of the genes in osteosarcoma. Firstly, cNLS-mapper [33] and TMHMM database [34] were used to analyze the gene sequencing of key genes and predict the nuclear localization regions and transmembrane domains. Then, Human Protein Atlas [35] which has been an effectively used online service for mapping certain proteins in various human cells, tissues and organs was used to explore the potential cellular location of the genes in osteosarcoma cells.

### Immunohistochemistry (IHC) experiment

#### Tissue samples

All the local hospital osteosarcoma samples used for tissue microarray production were all originally collected from Orthopaedics Department surgeries and stored in the hospital Biobank after being examined by Pathology Department, for ensuring the diagnosis and cancer percentage of the samples. Informed consent of the potential scientific application of the surgery samples have been obtained from patients at the same time they donated the samples to hospital Biobank, and the use of the tissues in the study was approved by the Hospital Institutional Board (Second Hospital of ShanXi Medical University, China).

In the study, 43 paraffin-embedded osteosarcoma patients samples (Table S4) were picked from hospital Biobank for IHC experiment after the reconfirmation of disease diagnosis and reevaluation of cancer percentage by pathology department using HE staining. Considering the high heterogeneity of osteosarcoma nature, instead of making the samples into tissue microarrays, the whole section of each tissue paraffin wax was used for the examination.

#### Equipment and regents

IHC experiment was performed using local hospital Biobank tissues to validate the differential expression of certain key gene in osteosarcoma comparing to adjacent normal tissues using local hospital Pathology Department instrument and equipment. The experiment was performed on VENTANA platform (Roche), the primary antibody of STC2 gene was purchased from proteintech (Cat No.60063-1-IG), the secondary antibody (Envision /HRP kit) and DAB detection kit were purchased from ZSBG-Bio by Immunohistochemistry Laboratory of Pathology Department, and other reagents including H2O2, phosphate-buffered saline (PBS), EDTA antigen retrieval citrate solution (PH = 8.8) and hematoxylin stain were all from hospital Supply Department.

#### IHC experimental protocol

The osteosarcoma samples paraffin slides were obtained from hospital Biobank and processed in Pathology Department, the slides were deparaffinized and rehydrated using gradient ethanol, then being treated with 0.3% H2O2 for inhibiting endogenous peroxidase activity followed by boiling in 10mmol/l EDTA citrate buffer for antigen retrieval. Further, the slides were soaked in bovine serum albumin for 20 min following incubating with primary STC2 antibody (dilution 1:200) overnight at 4 °C. Then after 20 min of rewarm at room temperature, the slides were incubated with specific secondary antibody at 37 °C for 1 h, next step being processed with horseradish peroxidase (HRP) and visualized in DAB with assistance of local hospital registered pathologists.

#### IHC results evaluation

The DAB staining result was evaluated with the assistance of two registered local hospital pathologists (Second Hospital of ShanXi Medical University, China) based on both the staining intensity and staining area. Any part of cell staining including membrane, cytoplasm and nuclear staining could be considered effective. The staining intensity was scored with the criteria set as: None (0), mild (1), moderate (2) and strong (3), meanwhile, the staining area was classified as: <1% (0), 1–25% (1), 26–50% (2), 51–75% (3) and > 75% (4).

The final score of each slide equals the multiplication of staining intensity and staining area, the eventual result would be regarded as negative with the multiplication value < 2, and the result would be defined as positive if the multiplication value ≥ 2, meanwhile, if the value was between 2 ~ 4, it was scored as “+”, 4 ~ 8 was “++” and when the multiplication value ≥ 8, it was defined as “+++”.

### Quantitative real-time PCR (qRT-PCR) experiments

To validate the changed expression of selected genes in osteosarcoma comparing to adjacent nomal tissues, the total mRNA of 20 paired of frozen stored osteosarcoma and adjacent normal bone tissues (20 of the 43 cases used for IHC experiment) were extracted using RNAiso-Plus (TAKARA, DaLian, China). Then1 µg extracted mRNA was used for cDNA synthesis using cDNA synthesis kit (TAKARA, DaLian, China). Further, qRT-PCR was performed on local hospital Pathology Department Roche z 480 equipment and the primers used were listed as below :

STC2:

Former: ATGCTACCTCAAGCACGACC.

Reverse: TCTGCTCACACTGAACC.

GAPDH:

Former: AGAAGGCTGGGGCTCATTTG.

Reverse: AGGGGCCATCCACAGTCTTC.

The qRT-PCR cycling procedure was set as: 95 °C 5 min for 1 cycle; 95 °C 5 s, 62 °C 30 s, and 72 °C 34 s for 35 cycles followed by the melting curve stage. The relative gene expression was calculated as the average of three replicates results based on 2^−ΔΔCT math algorithm. T-test was used for statistical analysis and P < 0.05 was considered statistically significant.

### Key gene expression in osteosarcoma comparing to other sarcomas and human cancers

Oncomine has been an effective web-based data mining platform which incorporates currently 715 independent datasets covering as much as 86,733 cancer samples for biomedical research data collecting, standardizing and analyzing [36]. In the study, Oncomine data was used to analyze and compare the expression of the selected key gene in pan human cancers, especially its expression in osteosarcoma when compares to other sarcomas and cancers.

### Key gene expression in osteosarcoma cells vs. other human cancer cell lines

CCLE, which is short for Cancer Cell Line Encyclopedia [37] has become a standard reference databases for cancer genomic analysis ever since its first cooperate foundation in 2012 by Broad Institute, Dana Farber Cancer Institute and Novartis biomedical Institute. The lastest version of the database covers the sequencing data of over 1457 human cancer cell lines from more than 30 tissue sources containing integrating genetic information including DNA mutation, gene expression, gene fusion, chromosome copy number and so on. In this study, CCLE database was used to compare the differential expression level of selected key genes between osteosarcoma and other cancer cell lines.

### Co-work genes mining and potential related signaling pathways analysis

To preliminary reveal the potential signaling pathways of the key gene’s regulation on osteosarcoma development, two different bioinformatic analysis tools were used. Firstly, STRING was used to construct the protein-protein interaction network and explore the surrounding genes that relate mostly with the selected key gene, following GO and KEGG annotating the signaling pathways that centered on the gene. Secondly, Oncomine was used for mining the co-expression genes relating to the selected key gene, and the gene’s potential biological functions were also preliminary explored.

## Results

### GEO data identified 815 high level differential expression genes in osteosarcoma comparing to normal bone samples

Based on the transcriptome data of the four GEO profiles, 12,168, 17,090, 9173 and 3062 genes were identified to be differential expressed in GSE12865, GSE16088, GSE28424 and GSE42352 respectively. Considering the heterogeneity nature of osteosarcoma between individuals from different research groups, we analyzed the four profiles genes separately. The genes of all four profiles were divided into four groups according to their expression level, namely the genes with expression discrepancy level < 2 fold, 2 ~ 4 fold, 4 ~ 8 fold and > 8 fold respectively (Table S2).

**Fig. 1:**
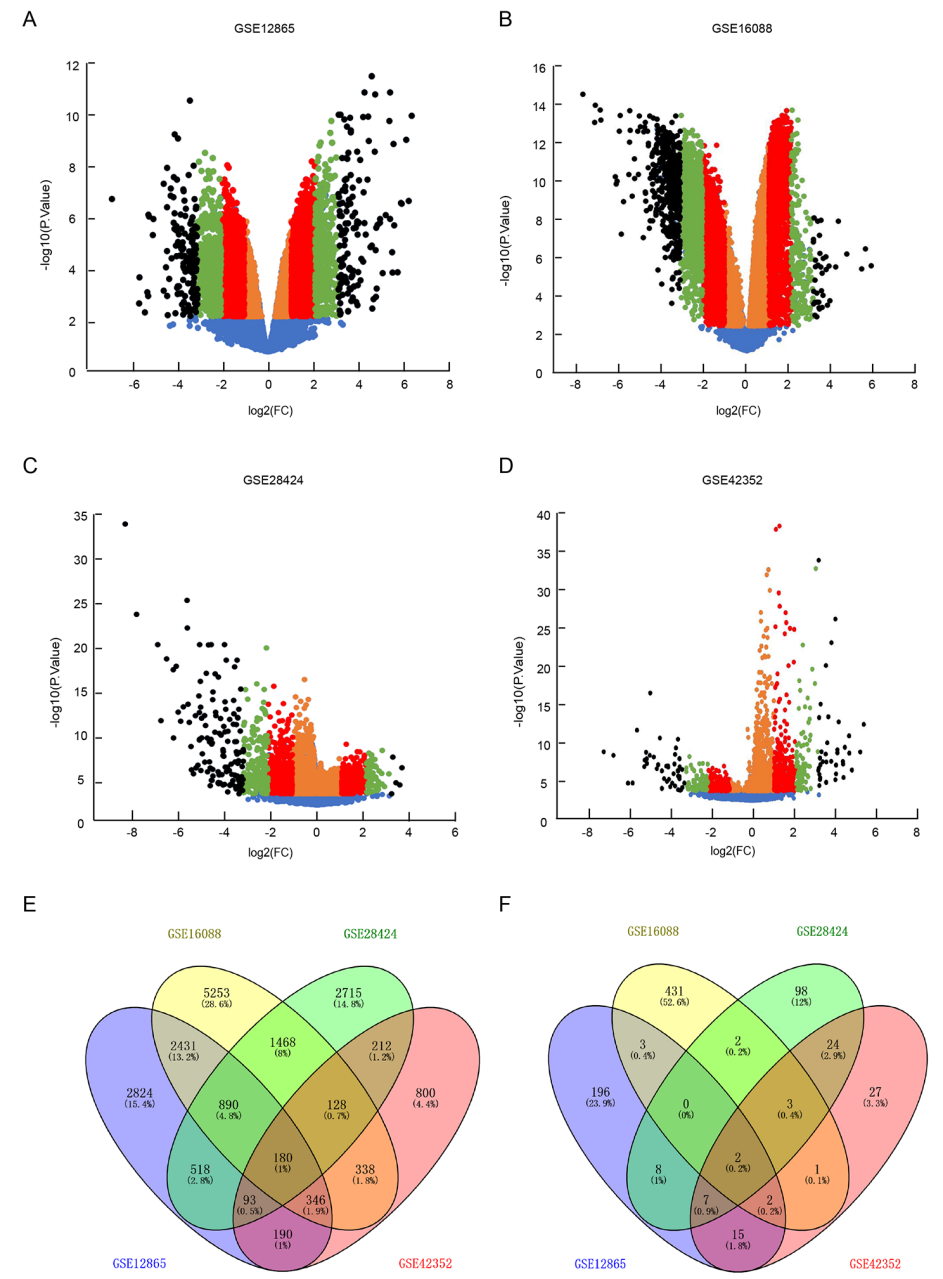
**The differential expression genes in osteosarcoma vs. normal control samples identified based on GEO datasets** From GEO datasets (A) GSE12865, (B) GSE16088, (C) GSE28424 and (D) GSE42352, the up-regulated (right-sided) and down-regulated (left sided) differential expression genes in osteosarcoma comparing to control samples were identified and classified into four groups based on difference level as: <2 fold genes(orange-colored spots), 2 ~ 4 fold genes (red-colored spots), 4 ~ 8 fold genes (green-colored spots) and > 8 fold genes (black-colored spots). (E) The intersection of all the differential expression genes in four GEO profiles. (F) The intersection of > 8 fold group of genes in four GEO profiles, the genes shared in at least two profiled were mainly focused for deeper analysis

And the results showed that in GSE12865, the expression change of 6050 genes were < 2-fold, 4864 genes were 2 ~ 4 fold, 962 genes were 4 ~ 8 fold and 292 genes were > 8 fold in osteosarcoma comparing to normal bone tissues (Fig. 1A). In GSE16088, the gene number was 9177, 5923, 1464 and 526 in each group respectively (Fig. 1B). In GSE28424, the gene number was 7543, 1194, 288 and 148 in each group (Fig. 1C). Meanwhile, in GSE42352, the gene number was 2286, 528, 166 and 82 in < 2 fold, 2 ~ 4fold, 4 ~ 8 fold and > 8 fold group respectively (Fig. 1D). A total of 10,974 DEGS were identified to be at least 2 fold differential expression in osteosarcoma comparing to normal control samples and 180 DEGS were shared in all four profiles. Meanwhile, the expression of 815 genes was > 8 fold, including 2 genes that were shared in all four profiles (Fig. 1E F, Table S2, S3).

### GO/KEGG interpretation of the differential expression genes revealed a specific location phenomenon

**Fig. 2:**
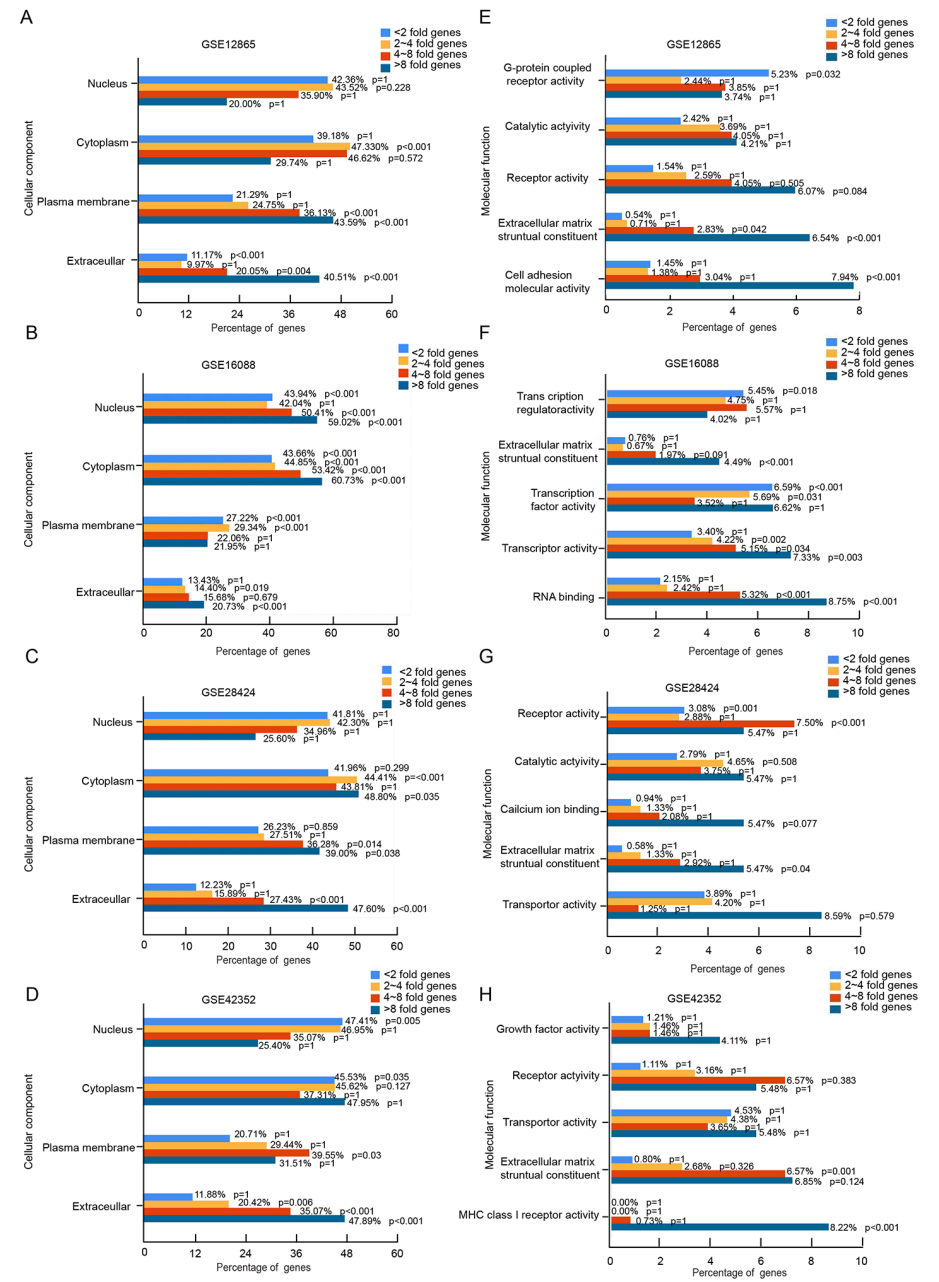
**GO/KEGG interpretation of the differential expression genes in osteosarcoma** The cellular components the differential expression genes (light blue colored bar represents the < 2 fold genes, orange bar represents 2 ~ 4 fold genes, red bar represents 4 ~ 8 fold genes, dark blue bar represents > 8 fold genes) mainly enriched in (A) GSE12865, (B) GSE16088, (C) GSE28424 and (D) GSE42352 respectively The molecular functions the differential expression genes (light blue colored bar represents the < 2 fold genes, orange bar represents 2 ~ 4 fold genes, red bar represents 4 ~ 8 fold genes, dark blue bar represents > 8 fold genes) mostly focused in (E) GSE12865, (F) GSE16088, (G) GSE28424 and (H) GSE42352 respectively

To preliminary interpret the biological functions of the differential expression genes and explore the potential difference among gene groups with diverse expression, GO and KEGG analysis were performed. And the result revealed an inspiring phenomenon that more different the genes expression are in osteosarcoma comparing to normal samples, their cellular location tend to be more outwards from the cell nuclear. To be more specific, the function analysis revealed that the cellular location of < 2 fold genes were mainly in nuclear, and 2 ~ 4 fold as well as 4 ~ 8 fold genes were mostly locating in the cytoplasm, meanwhile > 8 fold genes were tend to locate on the cell membrane and in extracellular region. Moreover, despite the genetic heterogeneity among different individuals, the phenomenon was shown in all four GEO patients profiles (Fig. 2A -2H). Considering the commonsense that a characteristic feature of osteosarcoma is the formation of specific osteoid matrix, we mainly focused on the > 8 fold genes which are not only more feasibly to be further tested using IHC experiment but also mainly locating in extracellular region for next step analysis.

### PPI network and function module analysis to screen the extracellular matrix construction related gene cluster

**Fig. 3:**
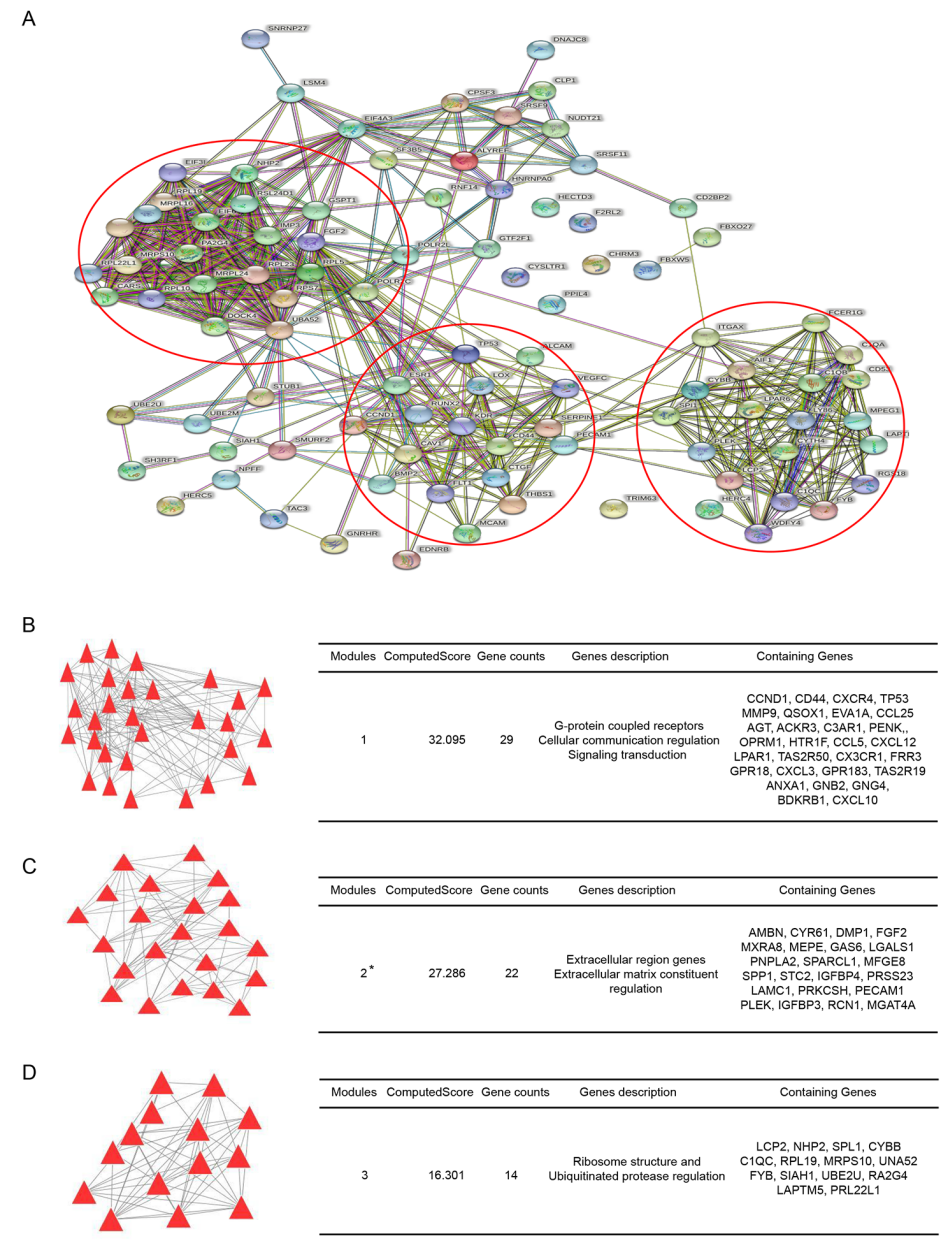
**Construction of PPI network of > 8 fold genes and function modules analysis** The PPI network of 67 > 8 fold genes which were shared in at least 2 GEO profiles and three genes modules were identified based on the net work (three red circles and each represents one gene module). The cyan lines between genes represent interaction predicted by known databases, light purple lines represent experimentally validated interactions, green lines mean neighborhood genes, red lines represent gene fusions, black lines represent genes with co-expression, meanwhile, the azure lines represent homology proteins. (B, C, D) The diagrammatic sketch (left diagram) and the detailed information (right table) including the module score, module description and detailed involving genes of three main modules in the PPI network. (*The 22 genes in second module were mainly focused for deeper analysis)

Based on the analysis of four GEO expression profiles, the expression of 292, 526, 148 and 82 genes were identified to be > 8 fold in GSE12865, GSE16088, GSE28424 and GSE42352 respectively, and 67 of the genes were identified in at least two of four profiles indicating they are more credible differential expression gene in osteosarcoma comparing to normal control samples. To further understand the correlation of the multiple genes and screen the potential key gene, PPI network of the 67 > 8 fold genes was constructed to firstly observe the interaction among individual genes and secondly to perform function module analysis thus exploring promising gene clusters that potentially relate with osteosarcoma extracellular matrix construction (Fig. 3A).

Eventually, three top gene clusters were identified based on the network, and the first module contains 29 genes which were mainly related with G protein coupled receptors and cellular communication (Fig. 3B). The second module contains 22 genes covering mostly extracellular locating and matrix construction related genes (Fig. 3C). And the third module includes 14 genes mainly associating with ribosome structure and ubiquitinated protease regulation (Fig. 3D). Given the specific potential connection between extracellular locating genes with the osteosarcoma specific osteoid matrix construction, the 22 genes in second module were highly payed attention for further survival analysis.

### Survival analysis identified STC2 as a promising risk indicator in osteosarcoma development

**Fig. 4:**
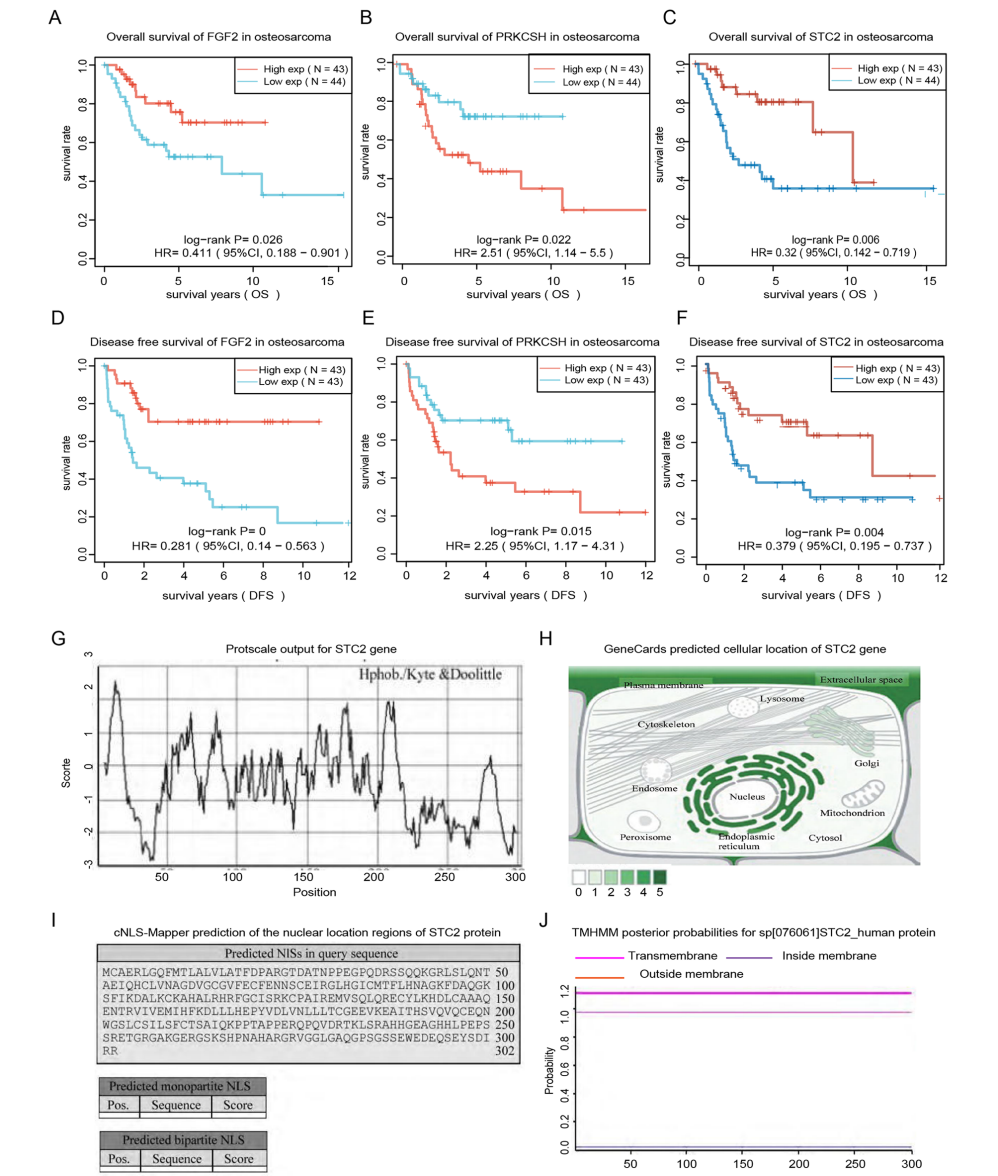
**Basic physicochemical properties analysis of STC2 gene** The overall survival analysis of (A) FGF2, (B) PRKCSH and (C) STC2 genes in osteosarcoma. Disease free survival of (D) FGF2, (E) PRKCSH and (F) STC2 genes in osteosarcoma. (G) The hydrophilcity / hydrophobicity analysis of STC2 protein. (H) The prediction model of the cellular location of STC2 protein. (I) cNLS-Mapper prediction of the nuclear location regions of STC2 protein. (J) TMHMM prediction of the transmembrane domains of STC2 protein

To further scale down the candidate genes and identify the promising responsible key gene during osteosarcoma development, KM survival analysis was performed using TARGET osteosarcoma transcriptome data to analyze the overall survival of the 22 extracellular matrix regulation related genes, and the result indicated that 3/22 genes were associated with both osteosarcoma overall survival and disease-free survival, namely FGF2 (Fig. 4A and D), PRKCSH (Fig. 4B and E) and STC2 (Fig. 4C and F).

GeneCards was then used to interpret the basic information of the three genes and the result revealed that STC2 is short for stanniocalcin 2 and has been reported to play a role in the regulation of cellular calcium and phosphate homeostasis, its over expression might result in reduced bone and skeletal muscle growth in mice models. And FGF2 is a member of the fibroblast growth factor (FGF) family and has been implicated in diverse biological processes, such as limb and nervous system development, wound healing, and tumor growth. Meanwhile, PRKCSH is an acidic phosphoprotein known to be a substrate for protein kinase C and related to the signaling pathways including small molecular transporting to cellular golgi and subsequent modification and innateimmune system.

Given the importance of the specific osteoid extracellular matrix formation in clinical diagnosis of osteosarcoma, we mainly focused on STC2 gene for further analysis.

### Basic physicochemical properties of STC2 gene

Before further exploring the detailed biological function of STC2 gene in osteosarcoma progression, ProtParam and ProtScale were combine used to interpret the basic physicochemical property of STC2 gene. And the results revealed that STC2, which is short for stanniocalcin2 locating in 5q35.2 encodes a protein composed of 302 amino acids including 35 positively charged amino acid residues (Arg + Lys) and 36 negatively charged amino acid residues (ASP + Glu). The molecular weight of the protein is estimated to be 33.2KD with the theoretical isoelectric point computed as 6.96.

Moreover, the protein half-life time of STC2 is computed to be 30 h in mammals and the estimated instability index is 38.27 indicating it works as a cellular stable protein. Meanwhile, ProtParam analysis computed the hydrophobic value of STC2 as 70.79 and average hydrophilicity as -0.518 which is consistent with ProtScale analysis which supported STC2 harbors several hydrophilic regions and shall be classified as a hydrophilic protein (Fig. 4G).

### Cellular location prediction of STC2 gene

Given the feasibility of further clinical medical testing of the gene by IHC experiment, cNLS-mapper, TMHMM database and Human Protein Atlas were in succession used to predict the cellular location of STC2 protein in human cells. And the result of cNLS-mapper analysis indicated that there’s no nuclear localization regions in STC2 protein (Fig. 4I), meanwhile, TMHMM database analysis ruled out the potential transmembrane domains existing in STC2 protein structure (Fig. 4J), followed by Human Protein Atlas analysis which revealed that STC2 mostly locates in cellular endoplasmic reticulum and extracellular regions (Fig. 4H).

To sum up above protein location analyzing and physicochemical properties predicting results which revealed that STC2 works neither through transmembrane nor nuclear locating dependent ways, it’s highly probable that STC2 works as a hydrophilic secretary protein in human cells and participates in signaling transduction related biological processes.

### STC2 expression is increased in osteosarcoma comparing to normal bone and other types of sarcomas

**Fig. 5:**
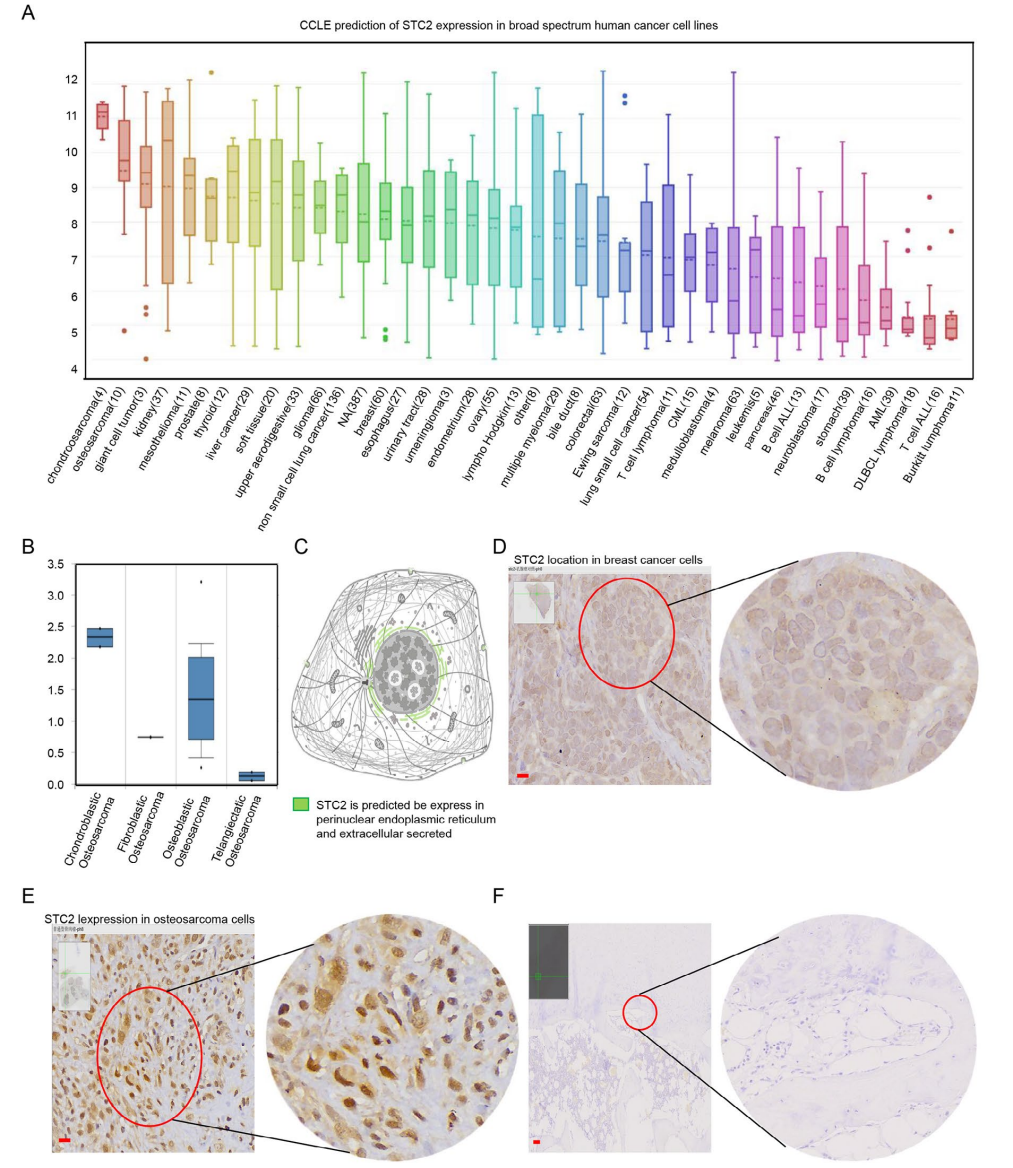
**Changed STC2 expression in osteosarcoma comparing to control samples** CCLE prediction of STC2 gene expression in broad spectrum human cancer cell lines, the number in abscissa parentheses represent count of cell lines involved in the analysis. (B) Oncomine compares the expression of STC2 in different osteosarcoma subtypes. (C) Human Protein Atlas predicts STC2 expresses in perinuclear endoplasmic reticulum or to be secreted extracellular. (D) IHC experiment using local hospital patients samples reveals STC2 expresses in perinuclear pattern as predicted in breast cancer which is suggested by the antibody operating instruction to be staining control cancer. (E) IHC experiment reveals that as opposed to the control breast cancer, most of the STC2 expresses in osteosarcoma as nuclear staining, and the expression was higher in cancer comparing to (F) control bone samples. Left corner of (D, E, F) was the original sample slice used for slide scanning. Scale bars, 20 μm. Magnification, 200x and 400x in the original images (left) and magnification images (right) for Figure D and E, meanwhile 50x and 400x for figure F respectively

To validate the differential expression of STC2 in osteosarcoma, bioinformatic analysis which was based on both cancer tissue samples and cell lines, as well as qRT-PCR experiment conducted using local hospital samples were conducted. Firstly, the analysis result of CCLE which has been a well known online database for cancer cell lines researches also supported that STC2 expresses much higher in chondrosarcoma and osteosarcoma cell lines comparing to other cancers and sarcomas (Fig. 5A).

Moreover, the Oncomine analysis result which database was constructed based on human tissue samples revealed that although STC2 expression various in multiple sarcomas, the expression was not only almost all up regulated in sarcomas including chondrosarcoma, liposarcoma, leiomyosarcoma, poorly differentiated sarcomas, osteosarcoma and so on comparing to normal tissues, but also the expression was the highest in osteosarcoma comparing to the other sarcomas. Meanwhile, as for the STC2 expression in different subtypes of osteosarcoma, the result indicated that it expresses higher in chondroblastic and osteoblastic osteosarcoma than fibroblastic and telangiectatic osteosarcoma (Fig. 5B).

Additionally, the result of qRT-PCR which was conducted using 20 local hospital osteosarcoma and paired normal bone tissues supported the STC2 gain of expression in cancer (data not shown).

### The association between STC2 gene and osteosarcoma clinical features

Besides the expression analysis based on online datasets and qRT-PCR experiment, the immunohistochemistry (IHC) experiment was also performed using local hospital patients samples. Firstly, the result verified the STC2 gain of expression in osteosarcoma comparing to normal osteoblasts based on the experiment fact that 41 of the 43 cancer samples showed positive staining (positive ratio 95.3%), meanwhile only 4 of 43 paired normal osteoblasts showed mild staining (staining ratio 9.3%).

Secondly, the association between STC2 expression and osteosarcoma clinical parameters was explored which result revealed that STC2 tends to expresses higher in patients with high Ki67 value and combined with tumor recurrence indicating it’s a potential disease risk indicator, meanwhile, no significance relationship was found between STC2 expression and patients age, gender, bone location, tumor volume nor necrosis (Table 1).

**Table 1:** The association between STC2 and osteosarcoma clinical pathological features

Parameters		STC2(%)		P Value
		- / +	++ / +++	
Gender				
	Male	11 (40.7)	16 (59.3)	0.555
	Female	8 (50.0)	8 (50.0)	
Age				
	< 20	8 (40.0)	12 (60.0)	0.861
	20 ~ 50	6 (46.2)	7 (53.8)	
	≥ 50	5 (50.0)	5 (50.0)	
Neoadjuvant chemotherapy				
	No	13 (38.2)	21 (61.8)	0.127
	Yes	6 (66.7)	3 (33.3)	
Tumor location				
	Left limbs	14 (56.0)	11 (44.0)	0.066
	Right limbs	5 (27.8)	13 (72.2)	
Necrosis				
	none	12 (37.5)	20 (62.5)	0.319
	< 10%	2 (66.7)	1 (33.3)	
	≥ 10%	5 (62.5)	3 (37.5)	
Tumor recurrence				
	No	16 (57.1)	12 (42.9)	0.019
	Yes	3 (20.0)	12 (80.0)	
Tumor Ki67 expression				
	< 14%	12 (66.7)	6 (33.3)	0.012
	≥ 14%	7 (28.0)	18 (72.0)	
Bone marrow affection				
	No	14 (45.2)	17 (54.8)	0.836
	Yes	5 (41.7)	7 (58.3)	
Distal metastasis				
	No	15 (48.4)	16 (51.6)	0.373
	Yes	4 (33.3)	8 (66.7)	

A fact worth emphasizing during the IHC experiment was that STC2 was predicted by Human protein Atlas to be normally express in perinuclear endoplasmic reticulum and extracellular regions (Fig. 5C), after ruling out the possibility of IHC technical elements based on the same experiment which supported that STC2 stains in perinuclear region in other cancers, for example breast cancer (Fig. 5D), the IHC experiment conducted on osteosarcoma samples showed that 38/41 cases were nuclear staining (Fig. 5E), meanwhile, the other 3/41 cases were membrane and cytoplasm staining, and mild or nearlly none obvious staining was observed in osteoblasts lining bone surfaces (Fig. 5F). Given above cNLS-mapper, TMHMM and Human Protein Atlas database predicting result which revealed that no nuclear localization regions nor transmembrane domains exist in STC2 protein structure, it is reasonable to infer that STC2 locates in octeosarcoma cellular nuclear through interacting with other elements, for instance nuclear locating transcription factors or regulators indicating a specific regulatory role of STC2 in osteosarcoma development.

### STC2 gene centered biological functions and related signaling pathways

**Fig. 6:**
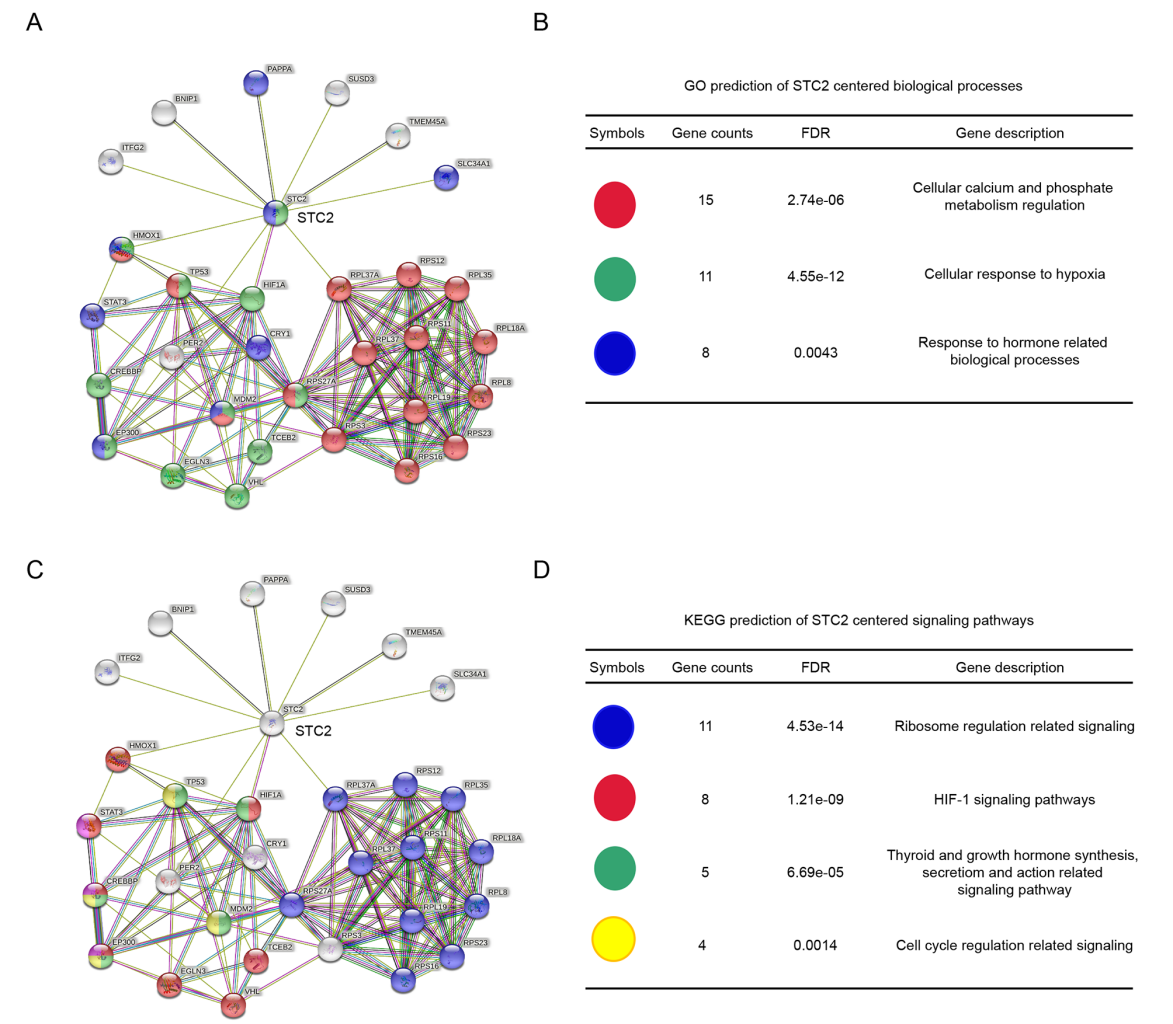
**STC2 centered biological functions and related signaling pathways** The PPI network which is centered on STC2 gene for analyzing (B) the main biological functions STC2 and its connected genes mainly participated in (C)The PPI network centered on STC2 for analyzing (D) the main involved signaling pathways In (A) and (C) diagram, the cyan lines represent the interaction between STC2 and responding genes were predicted by known databases, light purple lines represent interactions that were experimentally validated, green lines mean neighborhood genes, red lines represent gene fusions, black lines represent genes with co-expression, meanwhile, azure lines represent homology proteins as STC2. Preliminary interpretation of STC2 surrounding genes were listed in (B) and (D)

To preliminary explore the potential biological functions of STC2 gene in osteosarcoma and the probable signaling pathways involved, STRING was used to construct the STC2 centering PPI network for revealing the surrounding potentially connecting genes followed by GO and KEGG analyzing the probable signaling pathways these genes enriched in. And GO results showed that the biological processes STC2 gene participated in were mainly focused on cellular response to hypoxia, cellular calcium and phosphate metabolism regulation, response to hormone related biological processes (Fig. 6A and B).

Meanwhile, KEGG analysis revealed the signaling pathways FGF1 gene involved were mostly ribosome regulation related signaling pathway, HIF-1 signaling pathway, growth hormone synthesis, secretion and action related signaling pathway, as well as cell cycle modulation related pathways (Fig. 6C and D).

Above results shall provide promising directions for further exploring the mechanism of STC2 regulation on bone osteosarcoma development.

## Discussion

Osteosarcoma has been a common primary bone malignant tumor in children and adolescents, although the incidence is relatively low comparing to other human tumors, the degree of malignancy is threateningly high [38]. To make it worse, no notable improvement has been received for patients survival over the past 30 years, attributing partly to the clinical fact that neither of the emerging gene-targeting therapy nor immunotherapy which have been showing inspiring clinical effects in many other tumors receives encouraging response in osteosarcoma [39]. It is of clinical realistic value to keep exploring the genetic information of osteosarcoma thus screening potential responsible genes during cancer development and identifying promising gene indicators.

In current researches, the use of high throughput technologies for instance gene chips, microarrays and genome sequencing have been bringing in oceans of promising disease data [40–42], and a considerable part of these data are openly accessed to the public, making it more convenient for worldwide researchers to analyze the genetic events in cancer development and explore potential disease-causing gene alterations. In the study, multiple public online datasets and bioinformatic analyzing tools as well as local hospital biobank patients samples were combine used to explore promising genetic events in osteosarcoma development.

Firstly, four GEO transcriptome profiles of osteosarcoma namely GSE12865, GSE42352, GSE16088 and GSE28424 were together applied to analyze the differential expression genes in cancer comparing to control samples, which result revealed a cluster of clinical promising meanwhile changed expression genes in 4 profiles including 13,456 genes whose expression discrepancy was < 2 fold, 7984 genes with expression discrepancy 2 ~ 4 fold, 2175 genes with expression discrepancy 4 ~ 8 fold, and 815 genes whose expression discrepancy was > 8 fold in osteosarcoma versus control samples.

Next step GO/KEGG interpretation of the 4 groups of genes revealed a specific phenomenon that the more the genes’ expression discrepancy are, the more they tend to locate far away from cellular nuclear. To be more specific, the < 2 fold genes were shown to focus mainly in nuclear, and 2 ~ 4 fold as well as 4 ~ 8 fold genes were mostly located in cell cytoplasm, meanwhile the > 8 fold genes were prefer to locate on the cellular membrane or out in extracellular region. Similar phenomenon has been discovered in other cancers [43, 44] which makes reasonable sense considering the classic biology “central dogma” that most human functional proteins were synthesized in nuclear following DNA-RNA-protein direction, slight change in nuclear proteins which might play roles as transcription regulatory factors shall result in massive extracellular locating proteins’ expression difference [45, 46].

Considering the feasibility of further clinical validation using IHC experiment which has been one of the most commonly used pathology test, the more genes expression difference are, the more convenient the genes would be tested using IHC. Besides, in osteosarcoma, the extracellular matrix remodeling related genes have already been realized to play inspiring effects in cancer development [47]. Therefore, we paid high attention on the > 8 fold genes for next step analysis.

A total of 815 > 8 fold genes were identified in 4 profiles, and 67 of these 815 genes were shared in at least two profiles indicating they are more credible to be high level differential expression in osteosarcoma comparing to normal control samples. To further understand the genes’ relationship with each other, the PPI network of 67 high level DEGS was constructed followed by function module analysis. As a result, three top gene clusters involving multiple signaling pathways were identified based on the network, and of the three modules a 22 genes-containing cluster which were interpreted by GO/KEGG to be mostly extracellular matrix structural constituent regulation related genes drew our attention based on the commonsense that a characteristic feature of osteosarcoma is the formation of specialized osteoid matrix.

To further scale down the candidate genes and identify credible key genes during osteosarcoma development, multiple survival analysis was then in succession performed to evaluate the association between each of the 22 genes and osteosarcoma patients survival, and the result revealed three genes: STC2, FGF2 and PRKCSH, they were all supported to be associated with both patients overall and recurrence free survival. Given the GeneCards online interpretation of the three genes, we finally picked STC2 for further analysis for its reported function in the regulation of cellular calcium metabolism and bone microenvironment development.

STC2, which is short for stanniocalcin2, locates in 5q35.2 and encodes a hydrophilic protein composed of 302 amino acids including 35 positively charged amino acid residues (Arg + Lys) and 36 negatively charged amino acid residues (ASP + Glu), weighting 33.2KD and being predicted to be stable in human cells with estimated half-time as 30 h in all mammals. Meanwhile, cNLS-mapper and TMHMM online database analyzed that there’s no nuclear localization region nor transmembrane domain existing in STC2 protein structure, which is consistent with Human Protein Atlas and GeneCards prediction result that STC2 mainly locates in cellular endoplasmic reticulum and extracellular regions.

STC2 gene was selected base on previous GEO data exploration combined with further survival analysis which results supported that STC2 was one of the genes that were high level differential expression in osteosarcoma comparing to normal control samples and related with patients survival. To uniquely validate the gene’s expression change and to preliminary explore the gene’s regulation on osteosarcoma development, online databases analysis (Oncomine data) and IHC experiment conducted on 43 local hospital patients samples were performed.

The IHC experiment not only validated the gain of expression of STC2 in osteosarcoma (95.3%) comparing to adjacent control samples (< 10%), but also shown an inspiring fact that a big portion of samples were nuclear staining (92.7%) which is a different stain pattern than the previous location prediction result. STC2 has been reported to be differently expressed and play vital functions in many other cancers, for example breast cancer with a classic cellular location in erinuclear region [48, 49]. For ruling out the possibility of IHC technical elements, we conducted the IHC experiment with same reagent and equipment in breast cancer samples, and the result showed that STC2 stains as expected in perinuclear region in these samples. The changed cellular location in osteosarcoma indicated that STC2 participate in osteosarcoma development via specific mechanisms for example interacting with nuclear locating transcription factors or regulators which is certainly an inspiring direction for further research. Besides the IHC experiment, Oncomine online analysis also supported that although STC2 expression was broad spectrum up regulated in multiple sarcomas, the expression was higher in osteosarcoma than other tumors.

Inspired by above especially IHC experiment results, to preliminary explore the potential mechanism of STC2 regulation on osteosarcoma development, the PPI network of STC2 centered genes was constructed. Further, these surrounding genes were collected to analyze the probable signaling pathways they enriched in. And the result preliminary revealed that the biological processes STC2 gene participated in were mainly focused on cellular response to hypoxia, cellular calcium and phosphate metabolism regulation, response to hormone related biological processes. Meanwhile, the signaling pathways STC2 gene involved includes HIF-1 signaling pathway, growth hormone synthesis, secretion and action related signaling pathway, as well as cell cycle modulation related pathways.

Although previous study also reported the potential regulation of STC2 on osteosarcoma survival [50, 51], comprehensive and deeper analysis are still needed to confirm the regulatory role of STC2 in osteosarcoma development. Especially, although current GO/KEGG results shall provide an inspiring direction for next step revealing the detailed cellular function of STC2 gene in osteosarcoma development, detailed cell lines as well as animal models experiments are needed, more importantly, the STC2 surrounding genes are on the way to be analyzed in succession for exploring the potential responsible partner genes for STC2 specific location in osteosarcoma. The current result is not yet enough to classify the gene as an useful clinical drug target. We sincerely hope above results shall provide inspiring insight into better understanding of the disease and provoke worldwide researchers’ interest to further and deeper explore the bone malignancy and benefit the clinical treatment in near future.

## Conclusion

In conclusion, based on four GEO transcriptome profiles, we identified 24,430 genes that were differential expression in osteosarcoma comparing to normal control samples, and further series of bioinformatic analysis of the genes highlight STC2 as a valuable prognostic indicator in cancer development. Basic physiochemical properties of the gene was estimated and its gain of expression was validated using both online public data analysis and local hospital IHC experiment. Additionally, STC2 centered biological processes and signaling pathways were preliminary explored. Above results shall provide inspiring insights into better understanding the molecular mechanism behind osteosarcoma development.

## Electronic supplementary material

Below is the link to the electronic supplementary material.

Supplementary Material 1

## Data Availability

Publicly available datasets were analyzed in this study. The data can be found here: GSE12865:https://www.ncbi.nlm.nih.gov/geo/query/acc.cgi?acc=GSE12865 GSE16088:https://www.ncbi.nlm.nih.gov/geo/query/acc.cgi?acc=GSE16088. GSE28424:https://www.ncbi.nlm.nih.gov/geo/query/acc.cgi?acc=GSE28424. GSE42352:https://www.ncbi.nlm.nih.gov/geo/query/acc.cgi?acc=GSE42352. All data generated or analyzed based on the online datasets and other experiments during this study are included in this published article.

## List of abbreviations

DEGS: Differential expression genes
GEO: Gene Expression Omnibus
GO: Gene ontology
KEGG: Kyoto Encyclopedia of Gene and Genome
PPI: Protein-protein interaction network
STRING: Search Tool for the Retrieval of Interacting Genes
MCODE: Molecular Complex Detection
IHC: Immunochemistry experiment
qRT-PCR: Quantitative real time polymerase chain reaction
OS: Overall survival rate
DFS: Disease free survival rate
STC2: Stanniocalcin2
