# Supplementary material for: Osteosarcoma transcriptome data exploration reveals STC2 as a novel risk indicator in disease progression
Source: BMC Med Genomics. 2023 Feb 20;16:30. doi: 10.1186/s12920-023-01456-4 (PMC9942349; doi:10.1186/s12920-023-01456-4)
Supplement: Supplementary file 1 — Supplementary Material 1 [file 12920_2023_1456_MOESM1_ESM.pdf]

Supplementary Table 1 Detailed GEO datasets information used for identifying the candidate differently expressed genes in osteosarcoma vs control samples

GEO Dataset	Public data	Contributor	Sample amount	Accessing website
GSE12865	Sep 08, 2009	Sadikovic B	12 osteosarcoma and 2 control samples	https://www.ncbi.nlm.nih.gov/geo/query/acc.cgi?acc=GSE12865
GSE16088	Nov 04, 2009	Davis S, Paoloni M	14 osteosarcoma and 3 control samples	https://www.ncbi.nlm.nih.gov/geo/query/acc.cgi?acc=GSE16088
GSE28424	Oct 16, 2012	Namløs HM, Meza-Zepeda LA. et al	19 osteosarcoma cell lines and 4 control samples	https://www.ncbi.nlm.nih.gov/geo/query/acc.cgi?acc=GSE28424
GSE42352	Dec 19, 2012	Marieke Lydia Kuijjer	84 osteosarcoma and 3 osteoblast samples	https://www.ncbi.nlm.nih.gov/geo/query/acc.cgi?acc=GSE42352

Supplementary Table 2 Differently expressed genes number in osteosarcoma vs. normal control samples analyzed based on four GEO profiles

	<2 Fold	2~4 fold	4~8 fold	>8 fold	overall
**GSE12865**	6050	4864	962	292	12168
**GSE16088**	9177	5923	1464	526	17099
**GSE42352**	2286	528	166	82	3062
**GSE28424**	7543	1194	288	148	9173
**overall**	13456	7984	2175	815	24430

Supplementary Table 3 Detailed information of the differently expressed genes in osteosarcoma shared in different GEO profiles

	Differently expressed genes that were shared in all four GEO profiles								
Adjust P<0.05	CDC25B	RBPMS	BTK	PAWR	HRAS	FOLR2	CCNB2	ABCC5	TXNRD1
	KLC2	APOC1	CCDC51	MMP13	ARHGEF6	SPI1	SORBS2	CDH5	SAP18
	TIGD6	STEAP4	STC2	INPP5D	NT5DC3	TYROBP	AKR1C3	DRAP1	KBTBD4
	DDAH1	UGP2	LCP2	STAB1	C9orf78	ATIC	ABI3BP	GPR4	FYN
	ARHGAP 4	KITLG	NAT1	PPP2CA	ACOT7	MMP9	WAS	BCL2L1	SH2D3C
	RRAS2	KLHL7	HPS5	NUP98	DDOST	HSPA4	HIF1A	LAPTM5	NOTCH4
	LTBP2	PPA1	COMT	UBE2A	GALNT2	CXCR4	PECAM1	CSF2RA	TMC6
	MOCS3	SH3BP4	UBAP1	GTF2E2	PDK4	SLCO2B1	CLDN5	QRICH1	TRIP6
	HK1	MGAT4B	PDE1A	SOCS5	BPGM	SARS	MAPK6	SLA	GFPT1
	COPS8	GMFG	PML	WDR3	FLT1	GNG12	ADH1A	RGS20	MGST2
	NCBP2	CD300A	FGF7	PTK2B	SMAD2	PSMD5	MFNG	ARHGAP 25	ICA1
	ASB6	SPP1	RASSF2	ANAPC13	ISLR	PSMD12	GARS	ACLY	TOLLIP
	ASNS	RGS1	CALCRL	LY86	LHFP	STMN1	VPS33A	TRIM24	KIAA0101
	NOS3	DYRK3	DTL	GJA4	UBE2Z	ANXA2	EDNRA	PNMA2	PVR
	CXorf36	USP24	CRK	INVS	RNASE1	FCGR2A	GANAB	OGFOD1	ALOX5
	FPGS	ZDHHC24	KIF20A	CDC20	DGKE	MXRA7	MCFD2	ZBTB16	RNASE6
	C1QB	LMOD1	HMGA1	AIF1	STEAP1	TSPYL1	SLC7A11	GPR176	NPR3
	TOMM34	PLCL2	GDF3	RTN4	CD33	SLC7A7	C1orf54	COL4A3 BP	ERAL1
	C1QA	GJC1	SULF1	PLEK	GAS6	CCL5	SPARCL1	TOP2A	NCKAP1
	NFKBIB	MXI1	DNMT1	S100A4	PNPLA2	PRC1	ABCC3	PTRH2	CD163
>8 fold	SPP1	MMP9							

Supplementary Table 4 Clinical information of local hospital Osteosarcoma patients involved in IHC experiment

Parameters	N (%)
Gender	
male	27 (62.8)
female	16 (37.2)
Age	
<20	20 (46.5)
20~50	13 (30.2)
≥50	10 (23.2)
Neoadjuvant chemotherapy	
No	34 (79.1)
Yes	9 (20.9)
Tumor location	
Left limbs	25 (58.1)
Right limbs	18 (41.9)
Necrosis	
none	32 (74.4)
<10%	3 (7.0)
≥10%	8 (18.6)
Tumor recurrence	
No	28 (65.1)
Yes	15 (34.9)
Tumor Ki67 expression	
<14%	18 (41.9)
≥14%	25 (58.1)
Bone marrow affection	
No	31 (72.1)
Yes	12 (27.9)
Distal metastasis	
No	31 (72.1)
Yes	12 (27.9)
